# Supplementary figures and images for: Histone modification dynamics at H3K27 are associated with altered transcription of in planta induced genes in Magnaporthe oryzae
Source: PLoS Genet. 2021 Feb 3;17(2):e1009376. doi: 10.1371/journal.pgen.1009376 (PMC7886369; doi:10.1371/journal.pgen.1009376)

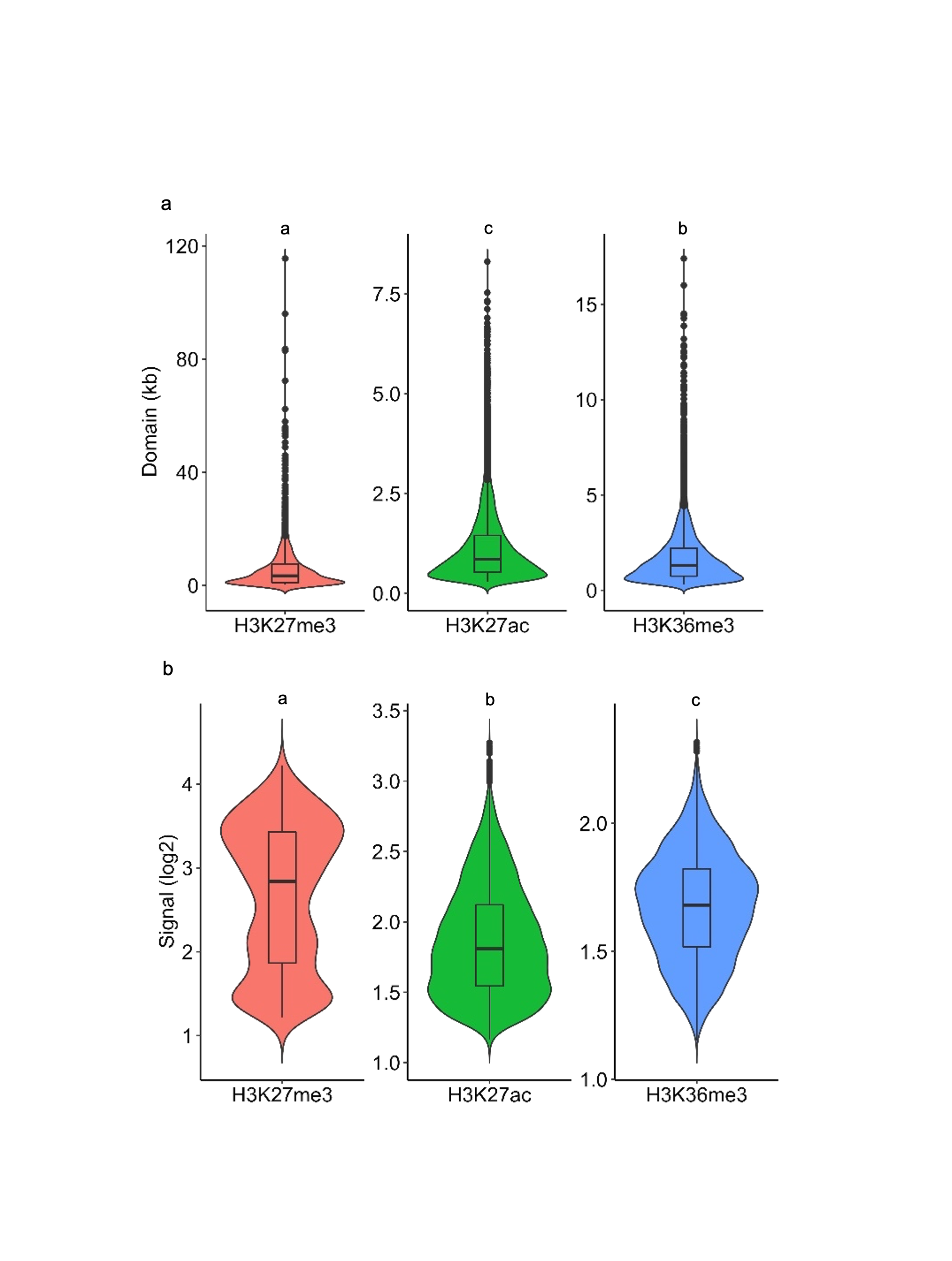

Supplement: S1 Fig — (A) and (B) Violin plots illustrating genome-wide distribution of domains (A) and ChIP signals (B) of H3K27me3, H3K27ac, and H3K36me3 in Guy11 wide type growing under in vitro complete medium. Letters above the violin plots indicates the significance. (TIF) [file pgen.1009376.s001.tif]

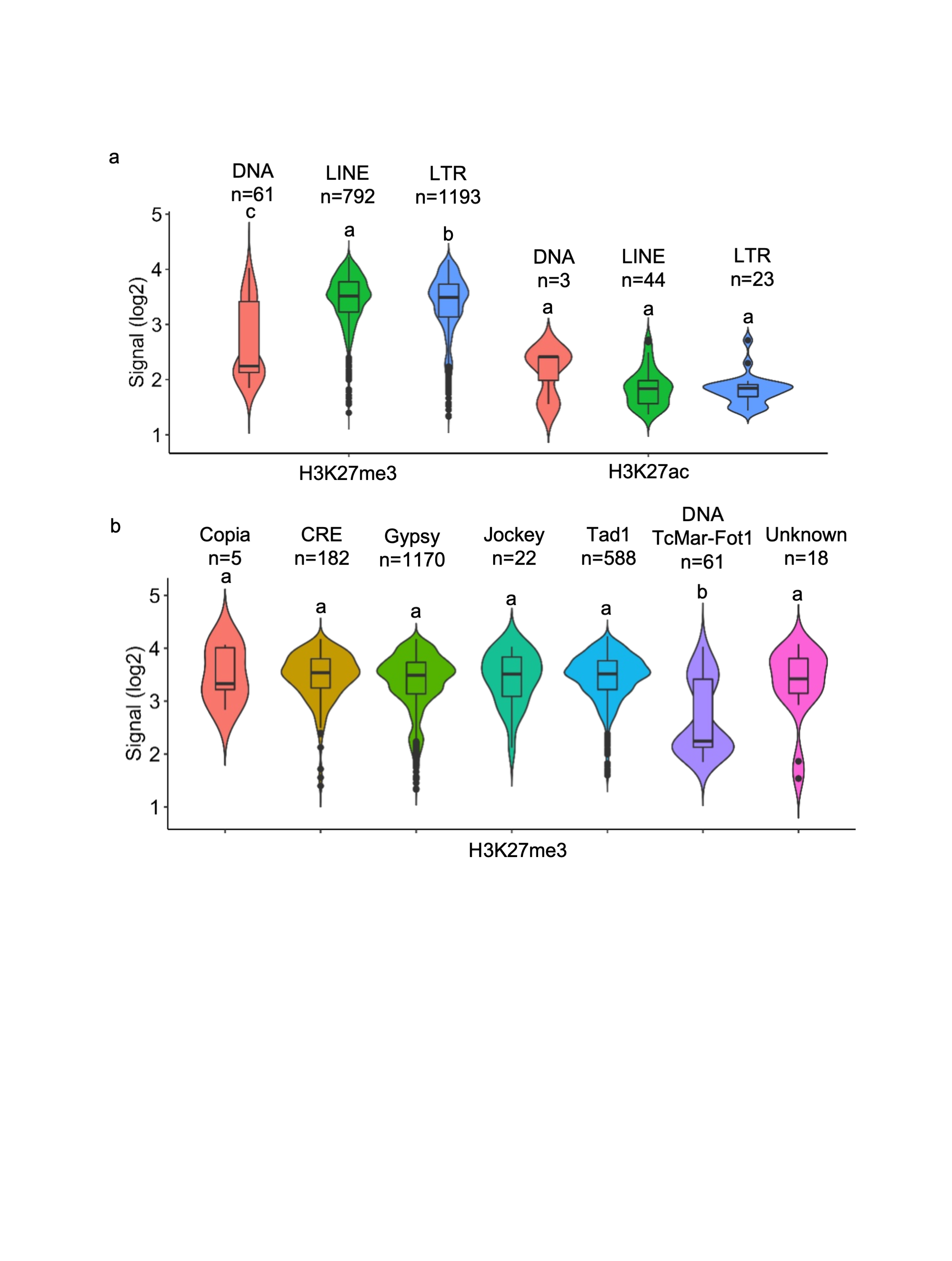

Supplement: S2 Fig — (A) and (B) ChIP signals of H3K27me3 and H3K27ac from MACS2 peak calling across transposable elements (TE) families (A) and subfamilies (B). ChIP-Seq data were collected from M. oryzae Guy11 wild type growing under in vitro complete medium. The number of TEs for each group is shown above each violin plot. Letters above the violin plots indicate the significant difference among groups based on ANOVA and Tukey’s HSD test. (TIF) [file pgen.1009376.s002.tif]

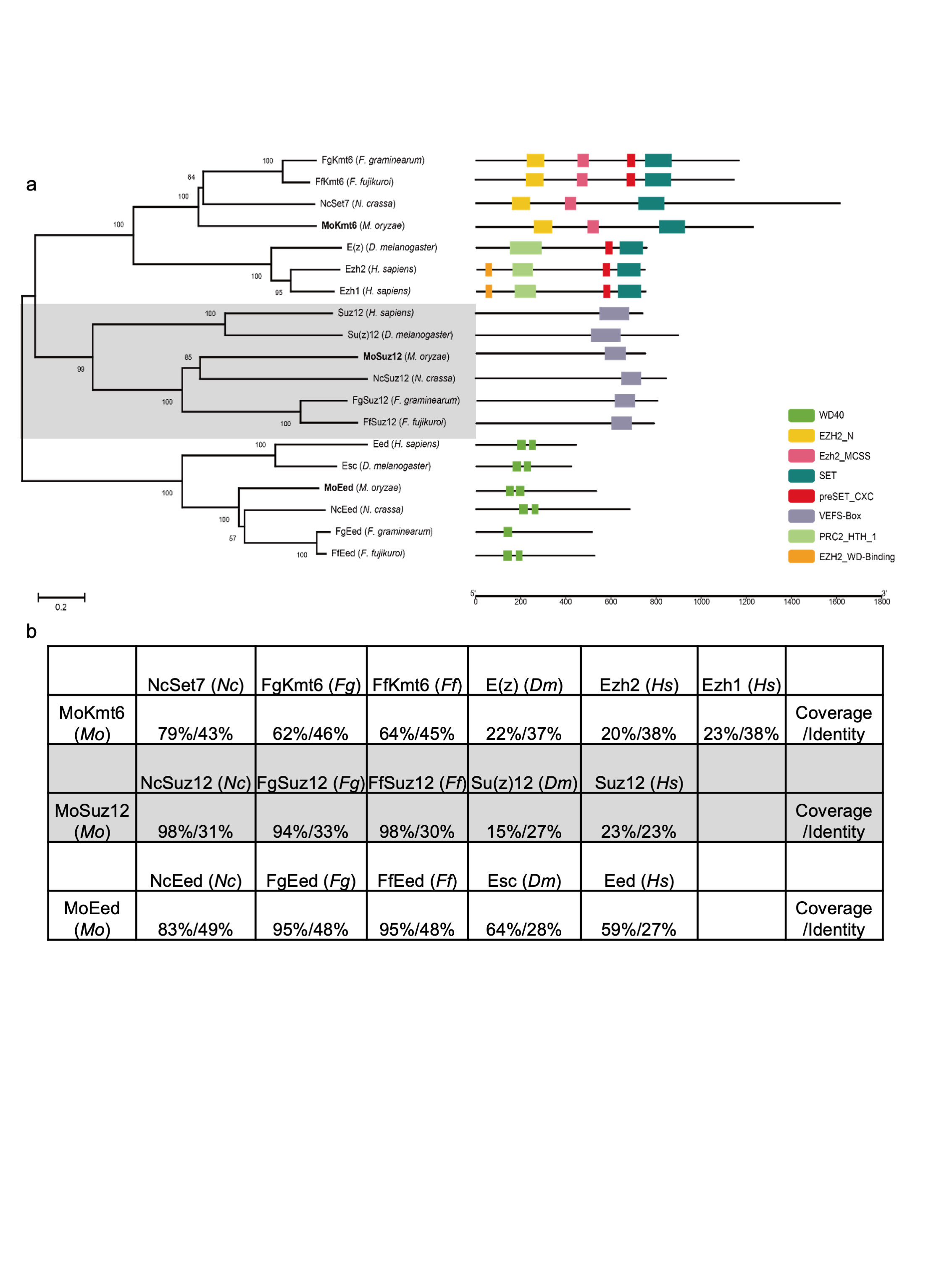

Supplement: S3 Fig — (A) Neighbor-joint tree of selected PRC2 core components generated by MEGA X with 1000 bootstrap replications. The protein domains are predicated by SMART and visualized by TBtools. The sequences used for analysis included MoKmt6 (MGG_00152), MoSuz12 (MGG_03169) and MoEed (MGG_06028) from M. oryzae; FgKmt6 (FGSG_15795), FgSuz12 (FGSG_04321), and FgEed (FGSG_15909) from F. graminearum; FfKmt6 (FFUJ_00719), FfSuz12 (FFUJ_09784) and FfEed (FFUJ_12272) from F. fujikuroi; NcSet7 (NCU07496), NcSuz12 (NCU05460), and NcEed (NCU05300) from N. crassa; Ezh2 (NP_001190176.1), Ezh1 (NP_001308008.1), Suz12 (NP_056170.2) and Eed (AAC23685) from H. sapiens; E(z) (NP_001137932.1), Su(z)12 (NP_730465.1), and Esc (NP_477431.1) from D. melanogaster. (B) Homology matrix analysis of M. oryzae PRC2 core components with PRC2 homologs in different organisms as described above. Numbers indicate protein coverage and identity. (TIF) [file pgen.1009376.s003.tif]

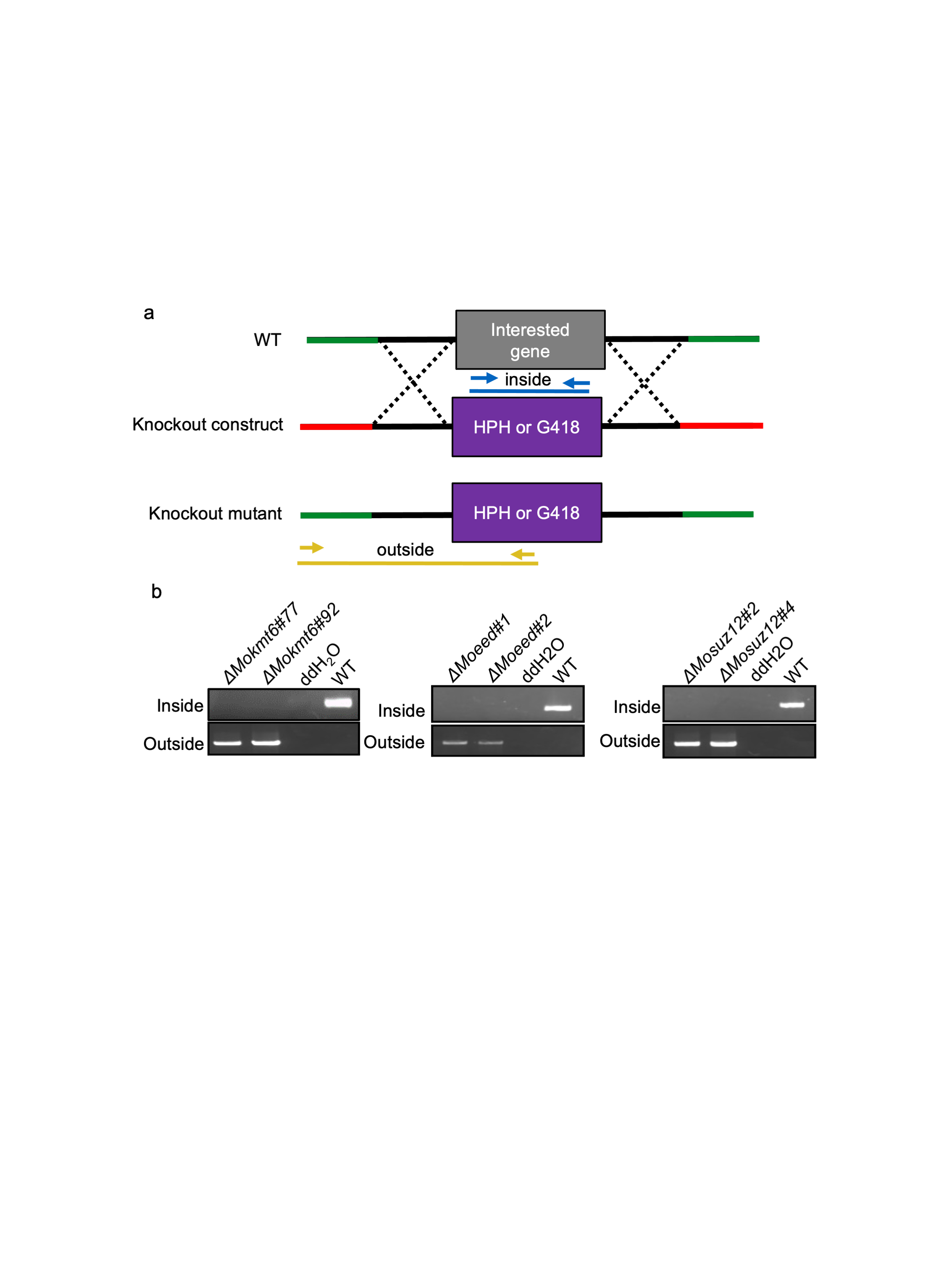

Supplement: S4 Fig — (A) Homologous recombination was used for gene knockout. ±1 kb gene flanking region (black lines) was amplified for targeting gene of interest. The gene coding region was replaced with hygromycin resistant cassette (HPH) for single knockout or geneticin resistant cassette (G418) for double knockout. Inside primer pair (blue arrow) was used for testing the presence/absence of gene of interest in transformants. Outside primer pair (yellow arrow) was used for testing whether there is correct resistant cassette integration in transformants. Green lines indicate upstream and downstream sequences of interested gene and red lines indicate upstream and downstream sequences of the resistant cassette. (B) ΔMokmt6, ΔMoeed, and ΔMosuz12 were confirmed by PCR amplifications with inside and outside primers. (TIF) [file pgen.1009376.s004.tif]

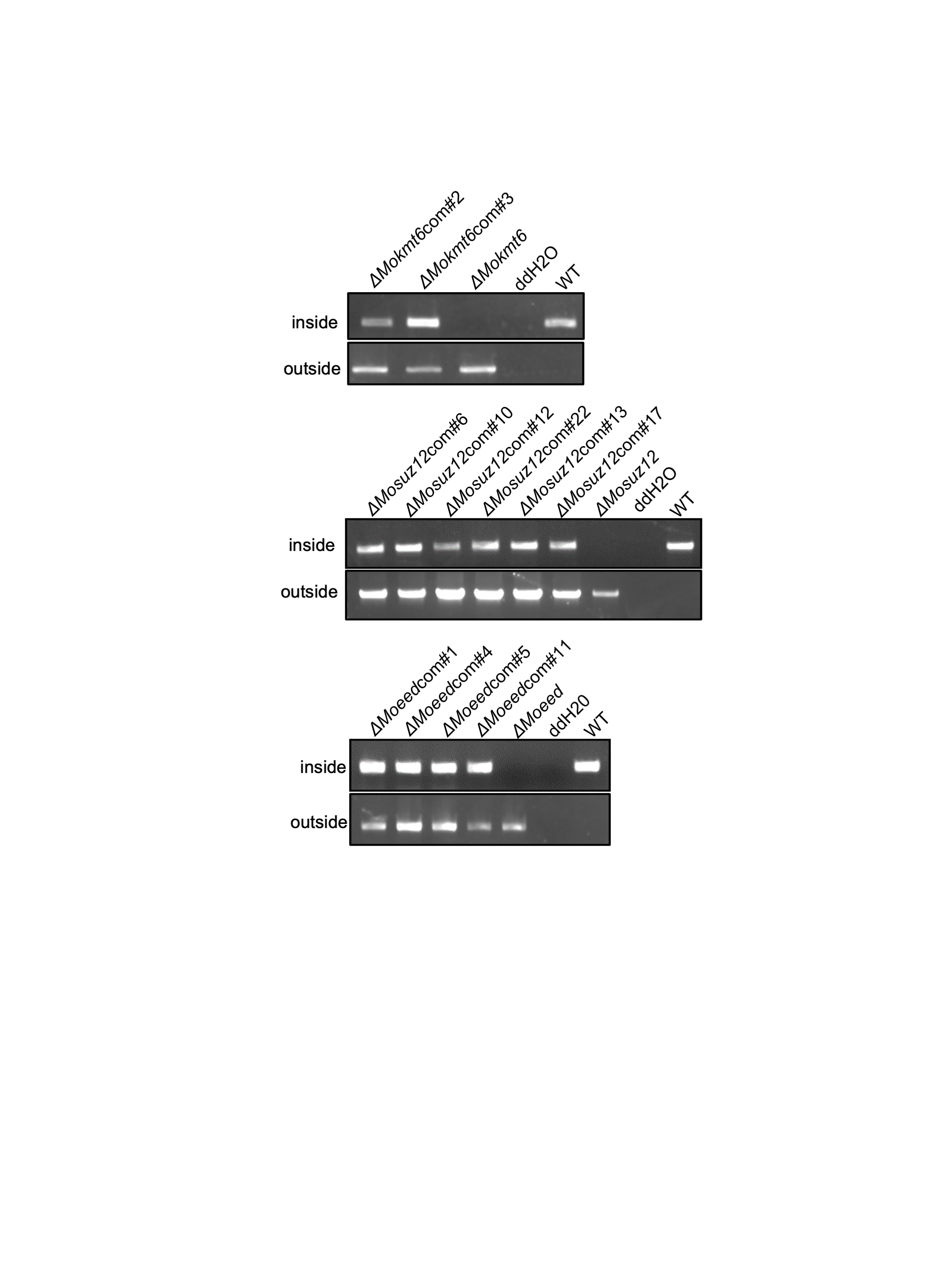

Supplement: S5 Fig — The complementation for ΔMokmt6, ΔMosuz12 and Δmoeed was screened by inside and outside primers. Amplifications with inside primers suggested the success in re-introducing the original gene back to the deletion mutants. (TIF) [file pgen.1009376.s005.tif]

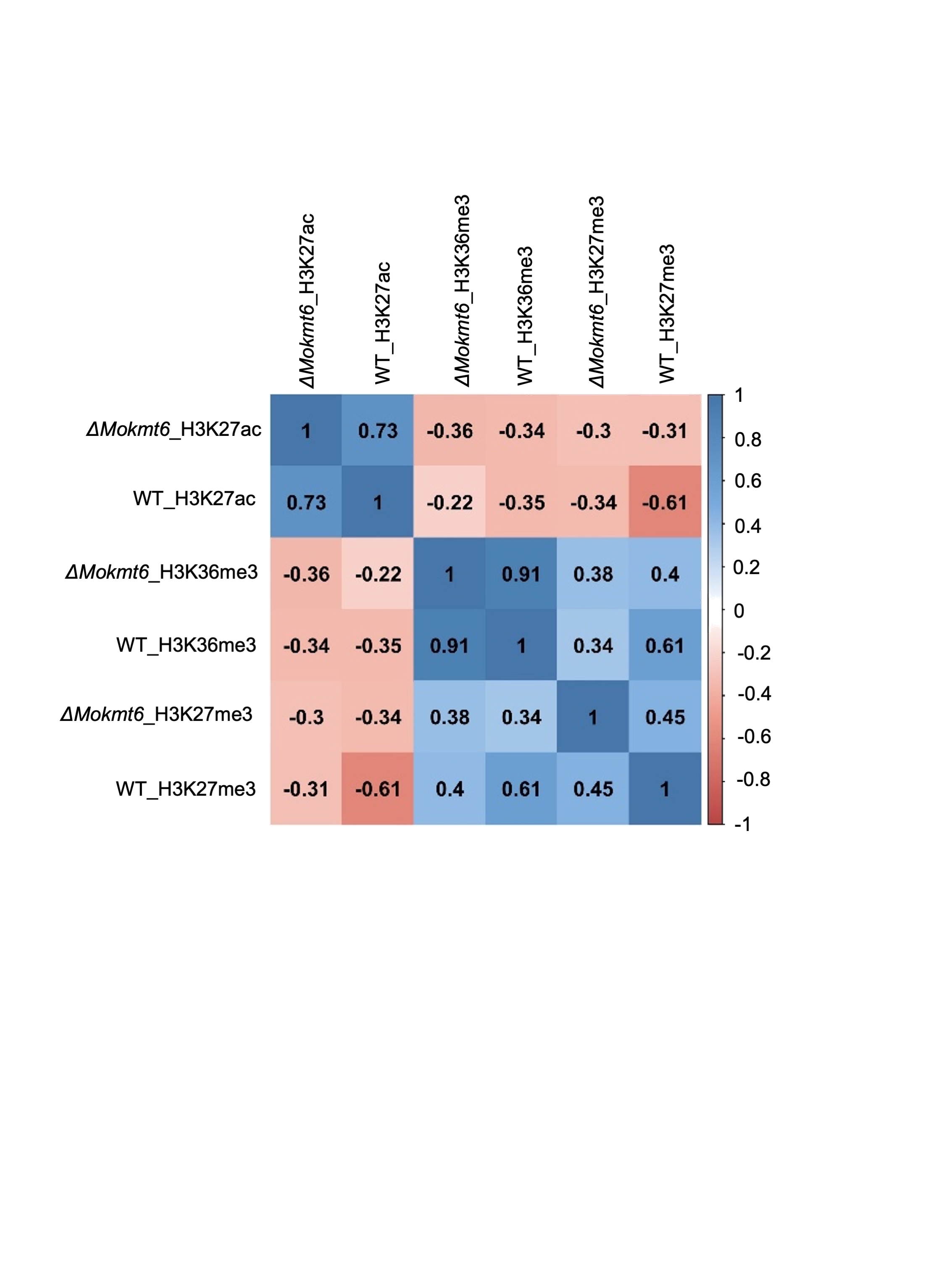

Supplement: S6 Fig — Heatmap illustrating pairwise Pearson correlation coefficiency of ChIP signals of H3K27me3, H3K27ac, H3K36me3 between Guy11 wild type (WT) and mutant ΔMokmt6 lacking H3K27me3. (TIF) [file pgen.1009376.s006.tif]

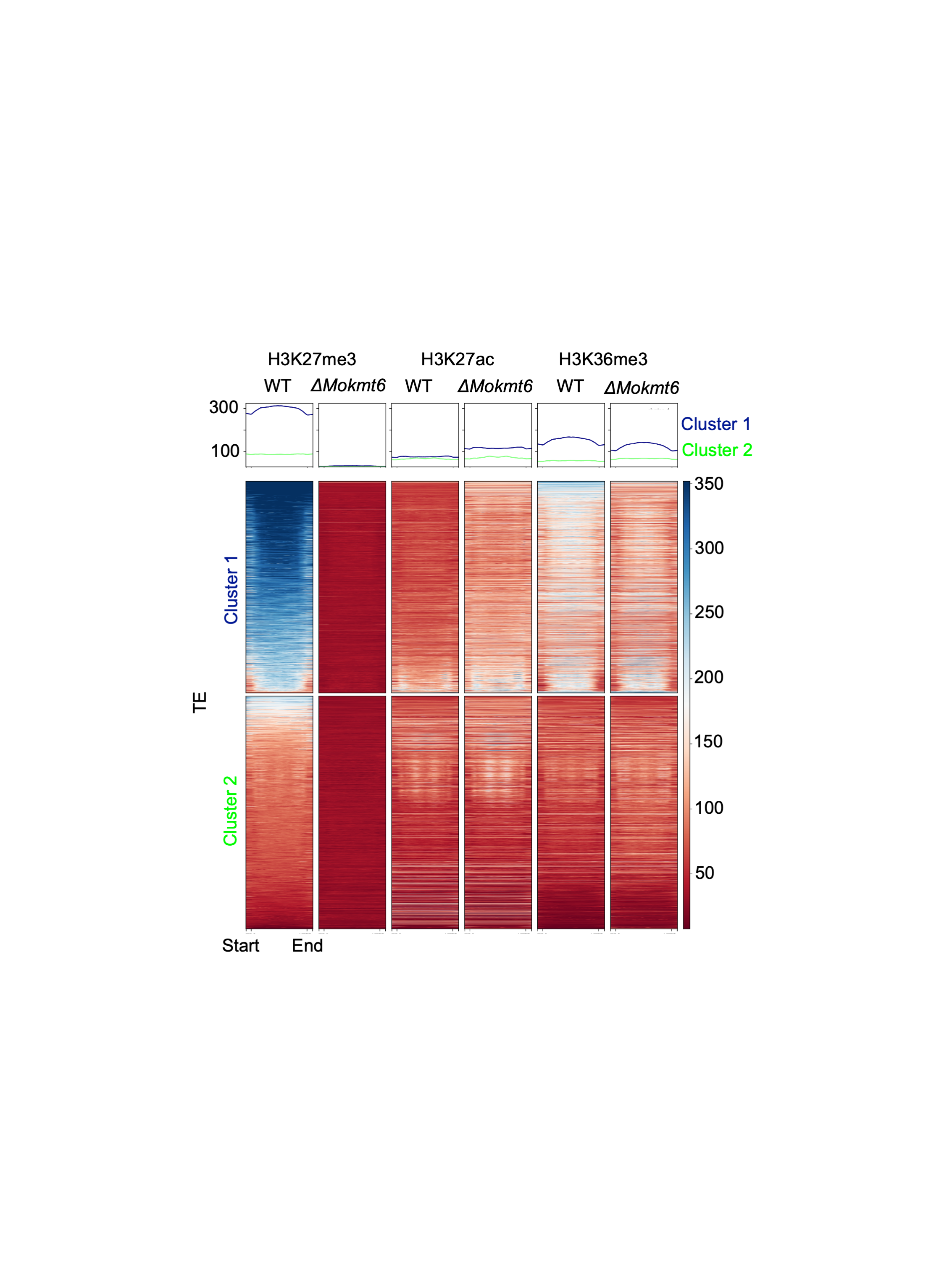

Supplement: S7 Fig — Heat maps visualizing profiles of ChIP signals for H3K27me3, H3K27ac and H3K36me3 across transposable elements (TEs) in Guy11 wild type (WT) and mutant ΔMokmt6. Two clusters of TEs are generated by unsupervised k-means analysis and ranked according to their H3K27me3 enrichment from wild type grown in vitro complete medium. (TIF) [file pgen.1009376.s007.tif]

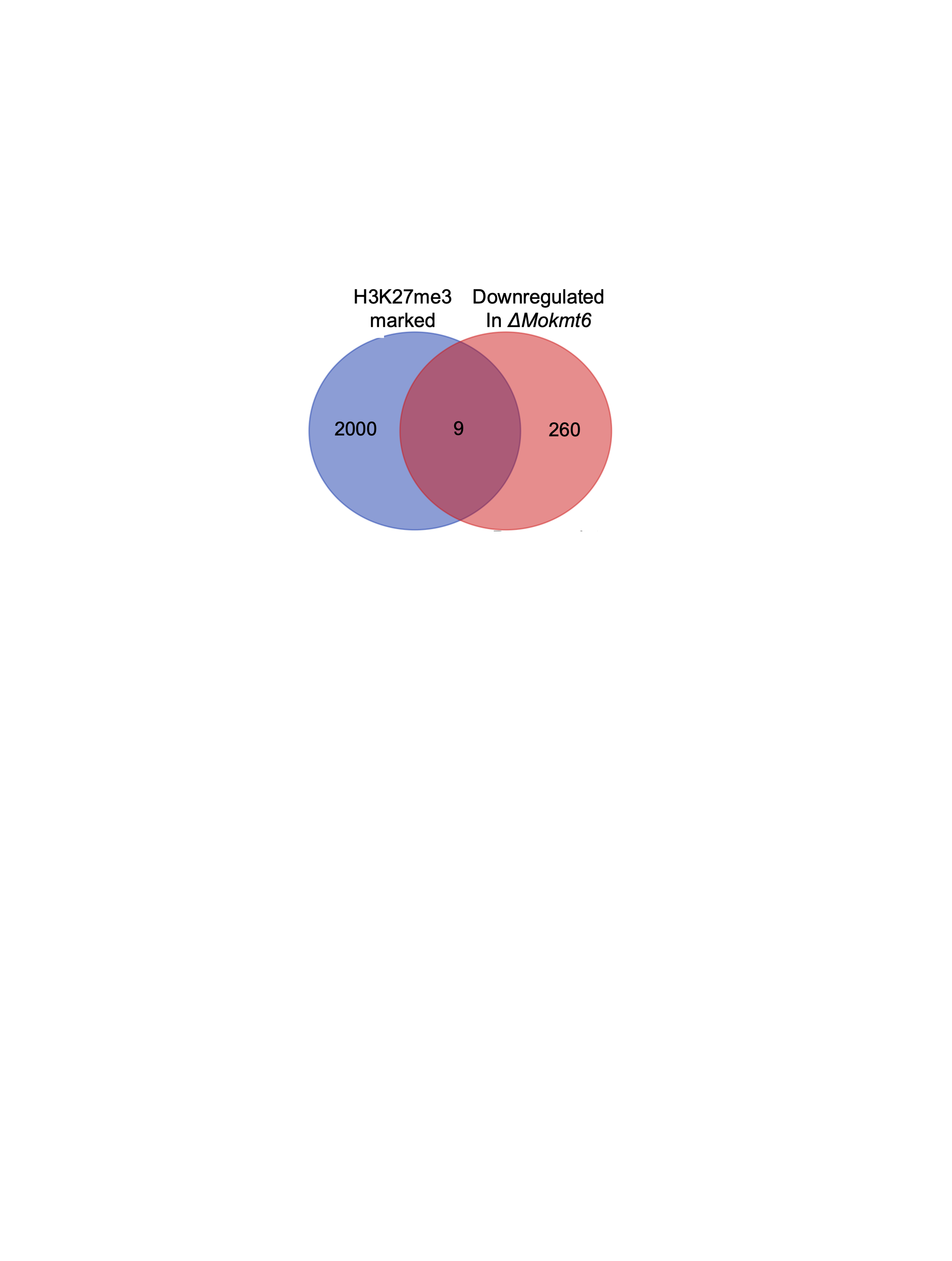

Supplement: S8 Fig — Venn diagram showing the overlap of genes downregulated in ΔMokmt6 and marked by H3K27me3 in Guy11 wild type. (TIF) [file pgen.1009376.s008.tif]

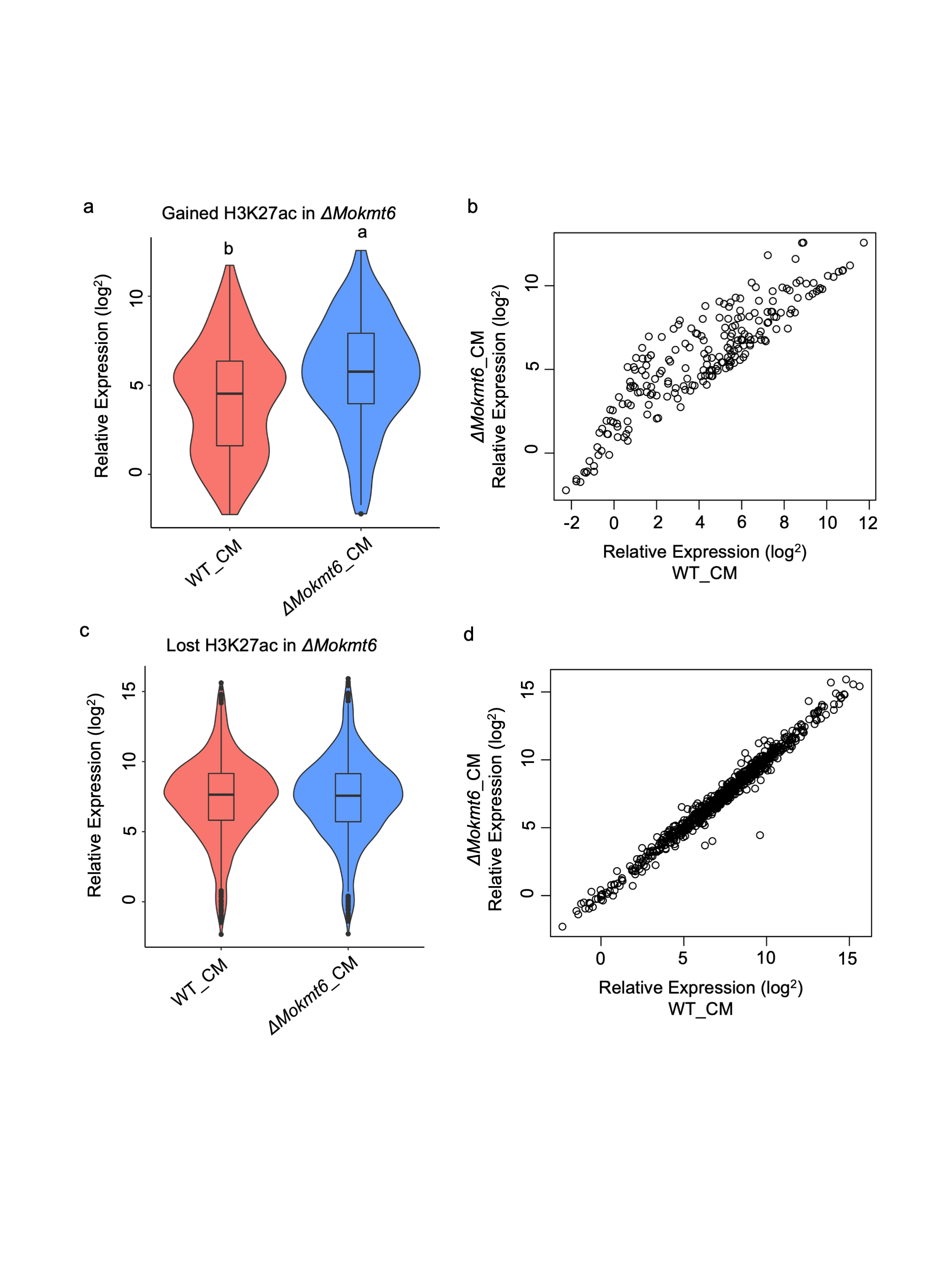

Supplement: S9 Fig — (A) Violin plot and (B) scatter plot showing gene expression profiles during in vitro complete medium (CM) growth in wild type and ΔMokmt6 for 218 expressed genes that gained H3K27ac in ΔMokmt6. (C) Violin plot and (D) scatter plot showing gene expression profiles during in vitro complete medium (CM) growth in wild type and ΔMokmt6 for 794 expressed genes that lost H3K27ac in ΔMokmt6. Letters above the violin plots indicate the significant difference among groups based on ANOVA and Tukey’s HSD test. (TIF) [file pgen.1009376.s009.tif]

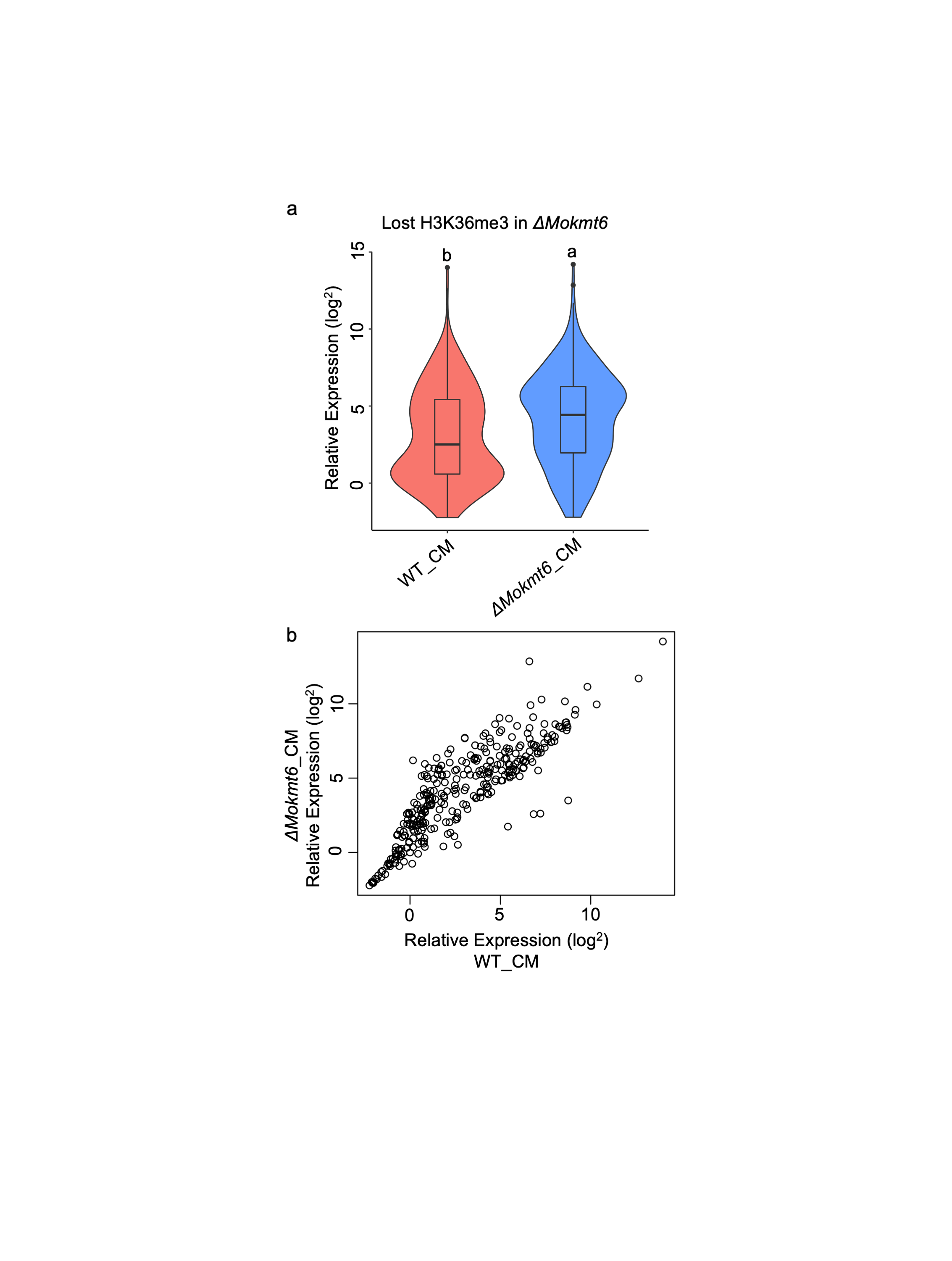

Supplement: S10 Fig — (A) Violin plots and (B) scatter plot showing the expression profiles during in vitro complete medium (CM) growth in wild type and ΔMokmt6 for 321 expressed genes that lost H3K36me3 in ΔMokmt6. Letters above the violin plots indicate the significant difference among groups based on ANOVA and Tukey’s HSD test. (TIF) [file pgen.1009376.s010.tif]

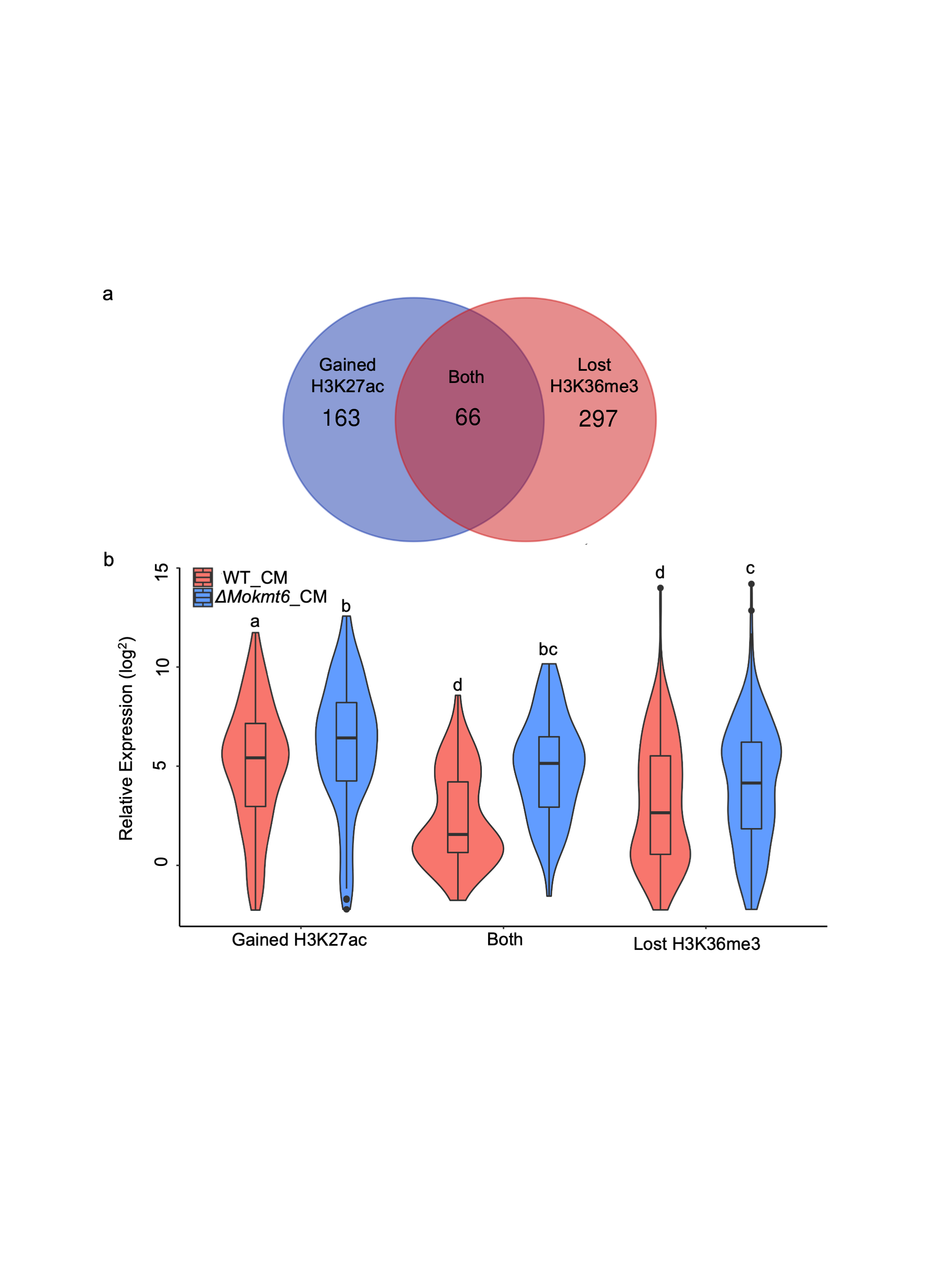

Supplement: S11 Fig — (A) Venn diagram showing the number and overlap of genes that only gained H3K27ac, only lost H3K36me3 or had both changes occur in the ΔMokmt6 mutant compared to wild type under in vitro complete medium (CM) growth. (B) Violin plots for the RNA-seq expression of wild type (WT_CM) and ΔMokmt6 (ΔMokmt6_CM) during in vitro complete medium (CM) growth for the gene sets from (A). Letters above the violin plots indicate the significant difference among groups based on ANOVA and Tukey’s HSD test. (TIF) [file pgen.1009376.s011.tif]

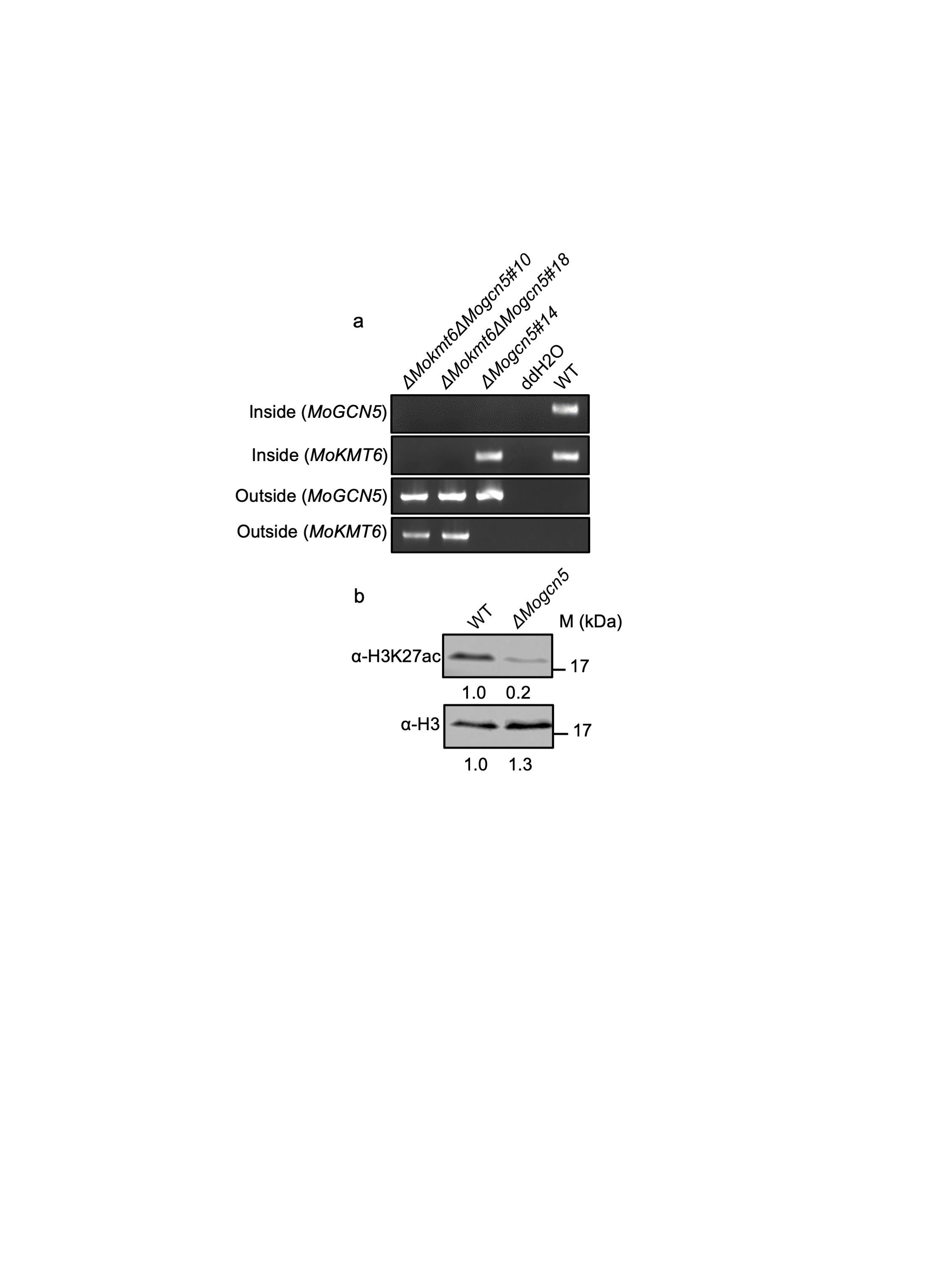

Supplement: S12 Fig — (A) ΔMogcn5 and ΔMokmt6ΔMogcn5 were confirmed by PCR amplification with inside and outside primers. (B) The anti-H3K27ac was used for detecting the global level of H3K27ac in the samples and anti-H3 was used as loading control. Signal intensities were measured by ImageJ. (TIF) [file pgen.1009376.s012.tif]

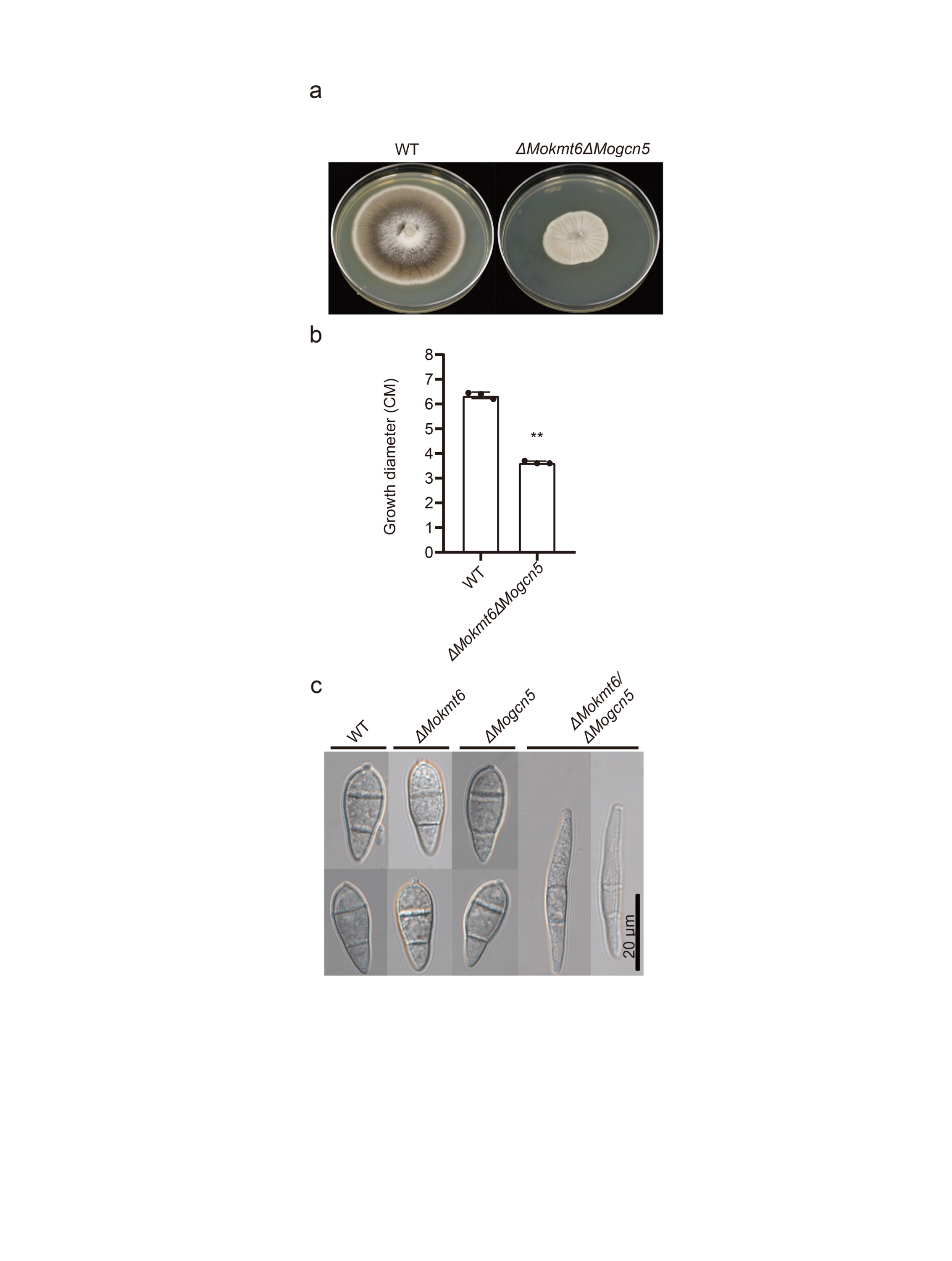

Supplement: S13 Fig — (A) Colony morphology of wild type and ΔMokmt6ΔMogcn5 on complete medium agar (CM) at 12 days. (B) Colony diameters measured at 12 days. The growth rates were determined to be significantly different (**, p <0.01) between wild type and ΔMokmt6ΔMogcn5 compared using student’s t-test. (C) Representative conidial morphology of wild type (WT), ΔMokmt6, ΔMogcn5, and ΔMokmt6ΔMogcn5 collected after growth on rice polish agar. Bar = 20 μm. (TIF) [file pgen.1009376.s013.tif]

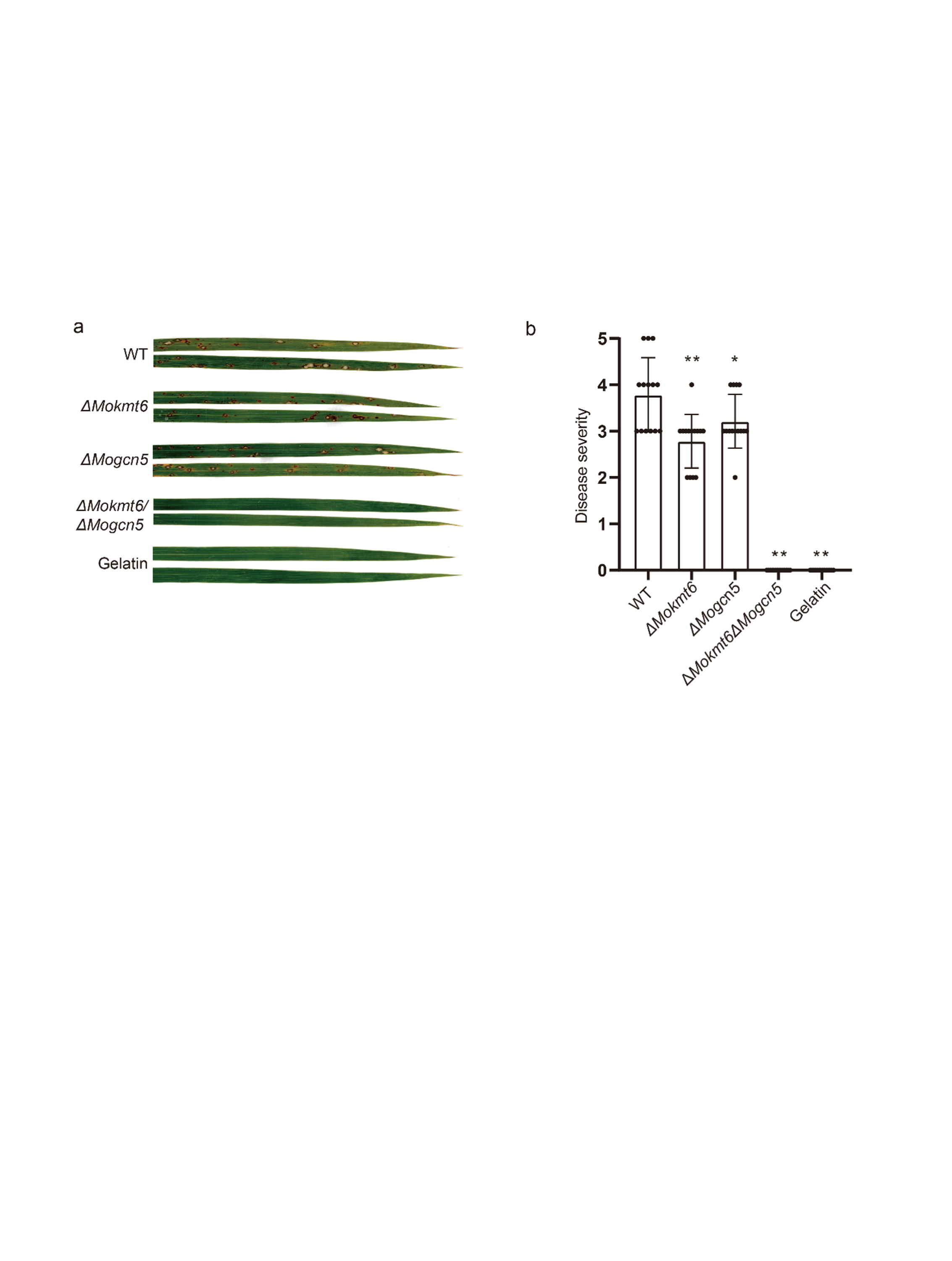

Supplement: S14 Fig — (A) Representative blast lesions of rice leaves sprayed with wild type (WT), ΔMokmt6, ΔMogcn5, ΔMokmt6ΔMogcn5 and 0.25% gelatin (control). The photos were taken at 7 days post inoculation and show typical blast lesions during a compatible interaction. (B) Bar plots showing quantitative analysis of 14 independent infected rice leaves (n = 14) from two biological experiments, classified as described in [68]. The disease severity rating for each treatment was compared to the wild type infection and student’s t-test was used to determine statistically significant differences. **, p <0.01; *, p <0.05. (TIF) [file pgen.1009376.s014.tif]

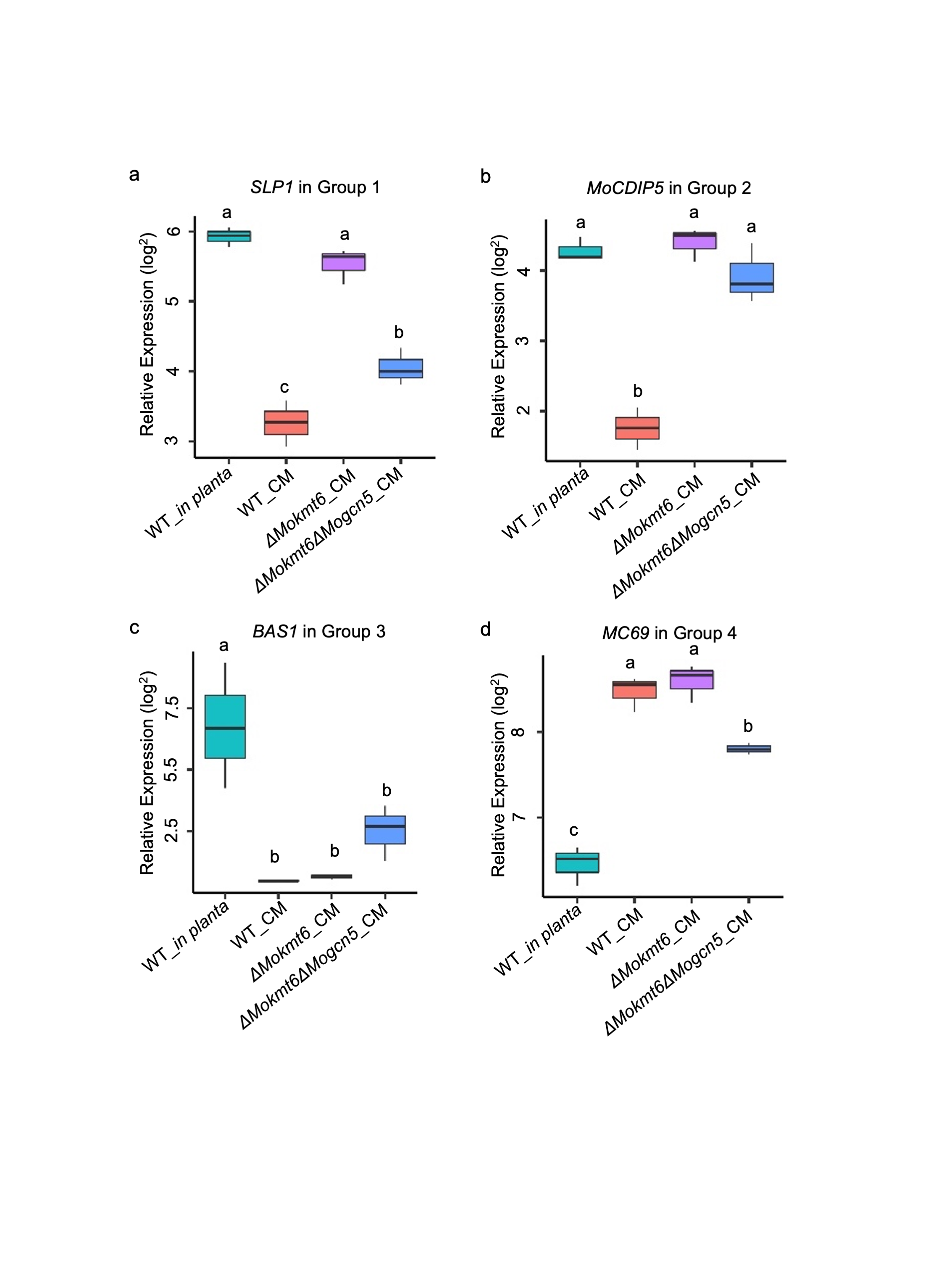

Supplement: S15 Fig — Expression of effector gene SLP1 (A), MoCDIP5 (B), BAS1 (C), and MC69 (D). RNA-seq data are collected from wild type (WT) M. oryzae strain Guy11 growing under in vitro complete medium (CM) and in planta, and two mutants ΔMokmt6 and ΔMokmt6ΔMogcn5 growing in CM. The mutant ΔMokmt6 lacks H3K27me3 and the double mutant ΔMokmt6ΔMogcn5 lacks H3K27me3 and majority of H3K27ac. Letters above the violin plots indicate the significant difference among groups based on ANOVA and Tukey’s HSD test. (TIF) [file pgen.1009376.s015.tif]

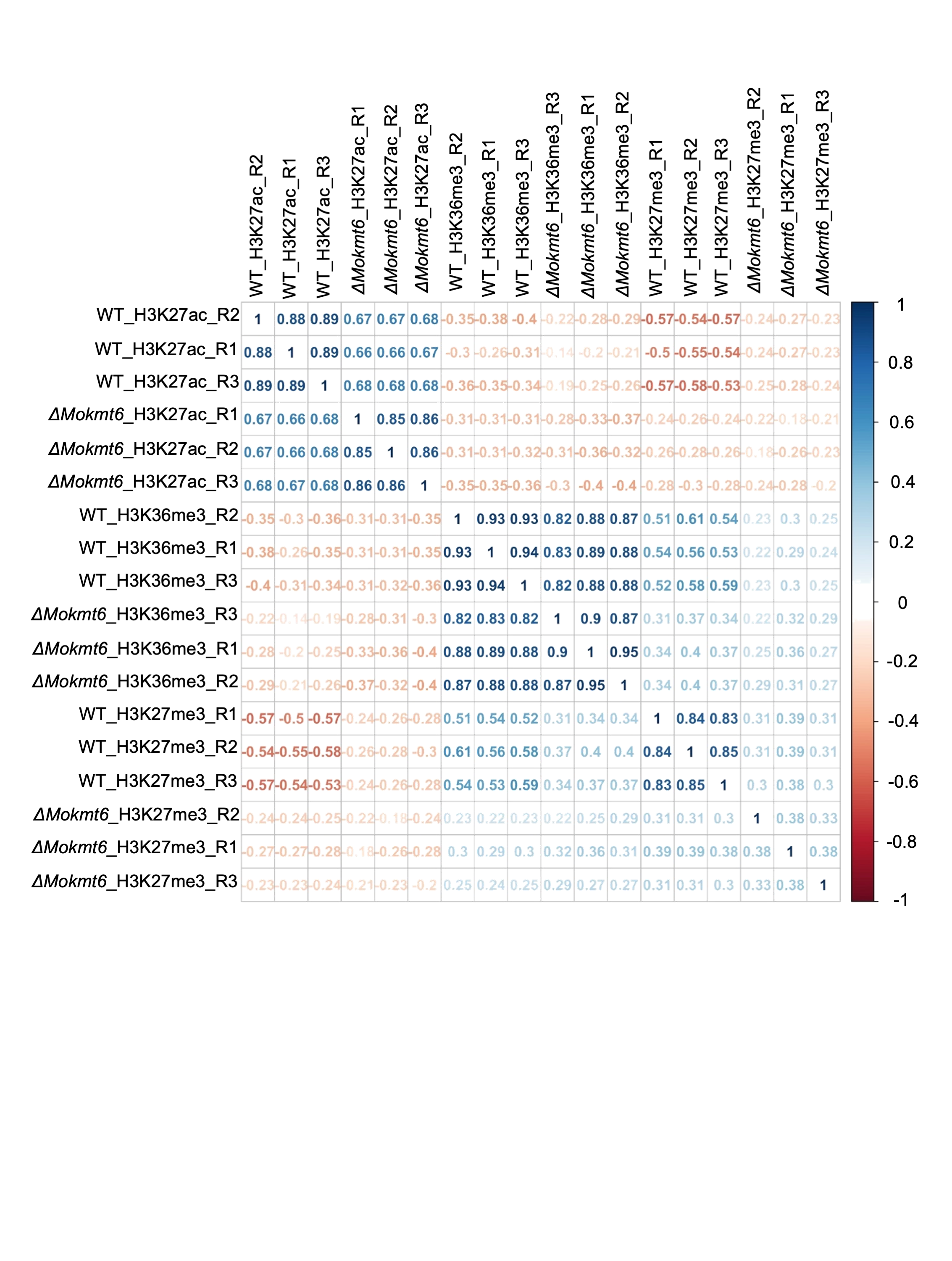

Supplement: S16 Fig — ChIP-seq experiment were conducted on M. oryzae Guy11 wild type (WT) and the mutant ΔMokmt6 with absence of H3K27me3 growing in complete medium (CM). R represents biological replicates. (TIF) [file pgen.1009376.s016.tif]

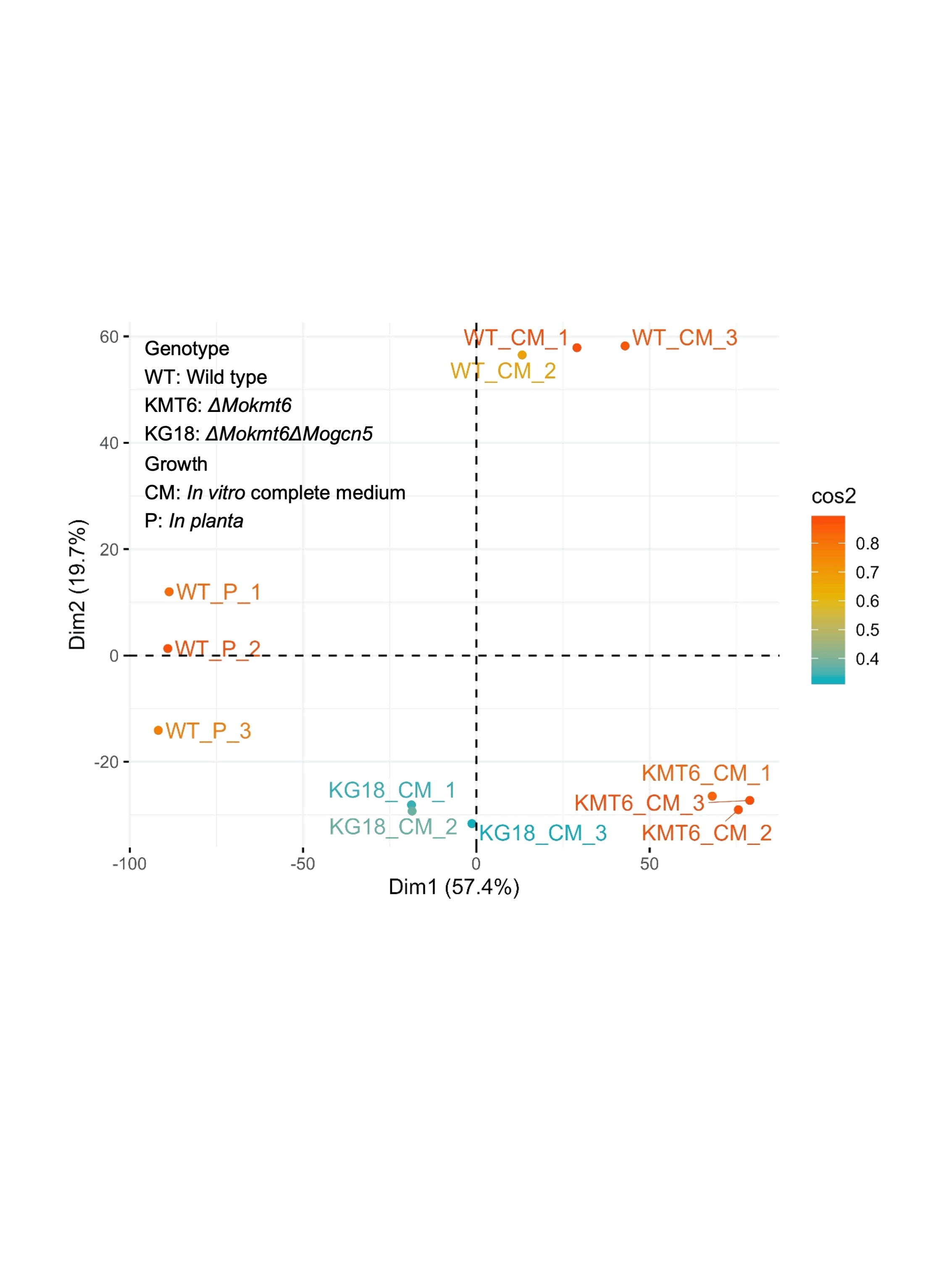

Supplement: S17 Fig — RNA-Seq experiment was conducted on RNAs extracted from M. oryzae Guy11 wild type (WT) growing under in vitro complete medium (CM) and in planta, and two mutants ΔMokmt6 and ΔMokmt6ΔMogcn5 growing in complete medium. The mutant ΔMokmt6 lacks H3K27me3 and the double mutant ΔMokmt6ΔMogcn5 lacks H3K27me3 and majority of H3K27ac. (TIF) [file pgen.1009376.s017.tif]
