# Supplementary material for: Histone modification dynamics at H3K27 are associated with altered transcription of in planta induced genes in Magnaporthe oryzae
Source: PLoS Genet. 2021 Feb 3;17(2):e1009376. doi: 10.1371/journal.pgen.1009376 (PMC7886369; doi:10.1371/journal.pgen.1009376)
Supplement: S9 Table — (DOCX) [file pgen.1009376.s026.docx]

**S9 Table. High-confidence characterized effectors, avirulence and *in planta* induced genes with H3K27me3 status.**

| Name | ID | Status_Guy11 | Gene ID | H3K27me3 Status | Group in Fig. 6a and b | Contig | Start | End | Length | References |
| --- | --- | --- | --- | --- | --- | --- | --- | --- | --- | --- |
| ACE1 | AJ704622 | Yes | gene00072 | Marked | 2 | contig_1 | 220404 | 232756 | 12353 | [[1](#_ENREF_1)] |
| Avr1CO39 | AF463528 | No Gene | NA | NA | NA | NA | NA | NA | 270 | [[2](#_ENREF_2)] |
| AvrPi54 | MGG_01947 | Yes | gene02661 | Marked | 2 | contig_11 | 1172651 | 1170643 | 2009 | [[3](#_ENREF_3)] |
| AvrPi9 | MGG_12655 | Yes | gene12077 | Marked | 1 | contig_8 | 2277558 | 2277217 | 632 | [[4](#_ENREF_4)] |
| AvrPiA | AB498873 | No Gene | NA | NA | NA | NA | NA | NA | 258 | [[5](#_ENREF_5)] |
| AvrPiB | KM887844 | No Annotation | NA | NA | NA | contig_5 | 88363 | 88587 | 225 | [[6](#_ENREF_6)] |
| AvrPii | AB498874 | No Gene | NA | NA | NA |  |  |  | 213 | [[5](#_ENREF_5)] |
| AvrPiK | AB498875 | No Annotation | NA | NA | NA | contig_13 | 82476 | 82817 | 342 | [[5](#_ENREF_5)] |
| AvrPiTA | AF207841 | No Function/Annotation | NA | NA | NA | contig_12 | 677569 | 676686 | 882 | [[7](#_ENREF_7)] |
| AvrPiZ-T | MGG_18041 | No Function | gene03135 | No | 2 | contig_13 | 880675 | 881001 | 327 | [[8](#_ENREF_8)] |
| BAS1 | MGG_04795 | Yes | gene06575 | Marked | 3 | contig_3 | 873,067 | 872720 | 348 | [[9](#_ENREF_9)] |
| BAS107 | MGG_10020 | Yes | gene05884 | Marked | 3 | contig_20 | 223251 | 223649 | 399 | [[9](#_ENREF_9)] |
| BAS162 | MGG_09379 | Yes | gene09294 | Marked | 3 | contig_5 | 783536 | 783979 | 444 | [[9](#_ENREF_9)] |
| BAS2 | MGG_09693 | Yes | gene04188 | No | 1 | contig_19 | 361929 | 362313 | 385 | [[9](#_ENREF_9)] |
| BAS3 | MGG_11610 | Yes | gene04466 | No | 3 | contig_2 | 883231 | 882706 | 526 | [[9](#_ENREF_9)] |
| BAS4 | MGG_10914 | Yes | gene09065 | Marked | 3 | contig_4 | 4395043 | 4395351 | 309 | [[9](#_ENREF_9)] |
| BAS52 | MGG_09378 | No Annotation | NA | NA | NA | contig_5 | 780821 | 781156 | 336 | [[9](#_ENREF_9)] |
| MC69 | MGG_02848 | Yes | gene11792 | No | 4 | contig_8 | 1307359 | 1307109 | 251 | [[10](#_ENREF_10)] |
| MGG_17227 | MGG_17227 | Yes | gene07700 | Marked | 1 | contig_3 | 4791702 | 4792127 | 426 | [[11](#_ENREF_11)] |
| MoCDIP1 | MGG_03356 | Yes | gene06941 | No | 3 | contig_3 | 2237737 | 2238804 | 1068 | [[12](#_ENREF_12)] |
| MoCDIP10 | MGG_12275 | Yes | gene00743 | No | 3 | contig_1 | 2188165 | 2187191 | 975 | [[13](#_ENREF_13)] |
| MoCDIP11 | MGG_12521 | Yes | gene11544 | No | 4 | contig_8 | 398162 | 397605 | 558 | [[13](#_ENREF_13)] |
| MoCDIP12 | MGG_13283 | Yes | gene04295 | Marked | 1 | contig_2 | 397176 | 396310 | 867 | [[13](#_ENREF_13)] |
| MoCDIP13 | MGG_14371 | Yes | gene09366 | No | 1 | contig_5 | 1036427 | 1035765 | 663 | [[13](#_ENREF_13)] |
| MoCDIP2 | MGG_05531 | Yes | gene03798 | No | 1 | contig_16 | 662927 | 662331 | 597 | [[12](#_ENREF_12)] |
| MoCDIP4 | MGG_08409 | Yes | gene00086 | Marked | 2 | contig_1 | 291548 | 290661 | 888 | [[12](#_ENREF_12)] |
| MoCDIP5 | MGG_10234 | Yes | gene03858 | Marked | 2 | contig_17 | 179535 | 180212 | 678 | [[12](#_ENREF_12)] |
| MoCDIP6 | MGG_01532 | Yes | gene01326 | No | 3 | contig_1 | 3939438 | 3938947 | 492 | [[13](#_ENREF_13)] |
| MoCDIP7 | MGG_03354 | Yes | gene06943 | No | 4 | contig_3 | 2241932 | 2241303 | 630 | [[13](#_ENREF_13)] |
| MoCDIP8 | MGG_05038 | Yes | gene05146 | No | 3 | contig_2 | 2721991 | 2720516 | 1476 | [[13](#_ENREF_13)] |
| MoCDIP9 | MGG_08411 | Yes | gene00088 | Marked | 3 | contig_1 | 298051 | 298644 | 594 | [[13](#_ENREF_13)] |
| MoNIS1 | MGG_02347 | Yes | gene03996 | No | 4 | contig_18 | 49131 | 48635 | 497 | [[14](#_ENREF_14)] |
| MoSVP | MGG_02778 | Yes | gene11865 | No | 4 | contig_8 | 1557230 | 1556589 | 642 | [[15](#_ENREF_15)] |
| MSP1 | MGG_05344 | Yes | gene03614 | No | 4 | contig_16 | 32779 | 32290 | 490 | [[16](#_ENREF_16)] |
| PWL1 | AB480169 | No Gene | NA | NA | NA | NA | NA | NA | 444 | [[17](#_ENREF_17)] |
| PWL2 | MGG_04301 | No Annotation | NA | NA | NA | contig_5 | 3047669 | 3048106 | 438 | [[18](#_ENREF_18)] |
| PWL2 | MGG_04301 | Yes | gene03367 | Marked | NA | contig_14 | 567097 | 567534 | 438 | [[18](#_ENREF_18)] |
| SLP1 | MGG_10097 | Yes | gene07702 | Marked | 1 | contig_3 | 4821119 | 4820539 | 581 | [[19](#_ENREF_19)] |

**References**

1. Bohnert HU, Fudal I, Dioh W, Tharreau D, Notteghem JL, Lebrun MH: **A putative polyketide synthase/peptide synthetase from Magnaporthe grisea signals pathogen attack to resistant rice.** *Plant Cell* 2004, **16:**2499-2513.

2. Ribot C, Cesari S, Abidi I, Chalvon V, Bournaud C, Vallet J, Lebrun MH, Morel JB, Kroj T: **The Magnaporthe oryzae effector AVR1-CO39 is translocated into rice cells independently of a fungal-derived machinery.** *Plant J* 2013, **74:**1-12.

3. Ray S, Singh PK, Gupta DK, Mahato AK, Sarkar C, Rathour R, Singh NK, Sharma TR: **Analysis of Magnaporthe oryzae Genome Reveals a Fungal Effector, Which Is Able to Induce Resistance Response in Transgenic Rice Line Containing Resistance Gene, Pi54.** *Front Plant Sci* 2016, **7:**1140.

4. Wu J, Kou Y, Bao J, Li Y, Tang M, Zhu X, Ponaya A, Xiao G, Li J, Li C, et al: **Comparative genomics identifies the Magnaporthe oryzae avirulence effector AvrPi9 that triggers Pi9-mediated blast resistance in rice.** *New Phytol* 2015, **206:**1463-1475.

5. Yoshida K, Saitoh H, Fujisawa S, Kanzaki H, Matsumura H, Yoshida K, Tosa Y, Chuma I, Takano Y, Win J, et al: **Association genetics reveals three novel avirulence genes from the rice blast fungal pathogen Magnaporthe oryzae.** *Plant Cell* 2009, **21:**1573-1591.

6. Zhang S, Wang L, Wu W, He L, Yang X, Pan Q: **Function and evolution of Magnaporthe oryzae avirulence gene AvrPib responding to the rice blast resistance gene Pib.** *Sci Rep* 2015, **5:**11642.

7. Orbach MJ, Farrall L, Sweigard JA, Chumley FG, Valent B: **A telomeric avirulence gene determines efficacy for the rice blast resistance gene Pi-ta.** *Plant Cell* 2000, **12:**2019-2032.

8. Li W, Wang B, Wu J, Lu G, Hu Y, Zhang X, Zhang Z, Zhao Q, Feng Q, Zhang H, et al: **The Magnaporthe oryzae avirulence gene AvrPiz-t encodes a predicted secreted protein that triggers the immunity in rice mediated by the blast resistance gene Piz-t.** *Mol Plant Microbe Interact* 2009, **22:**411-420.

9. Mosquera G, Giraldo MC, Khang CH, Coughlan S, Valent B: **Interaction transcriptome analysis identifies Magnaporthe oryzae BAS1-4 as Biotrophy-associated secreted proteins in rice blast disease.** *Plant Cell* 2009, **21:**1273-1290.

10. Saitoh H, Fujisawa S, Mitsuoka C, Ito A, Hirabuchi A, Ikeda K, Irieda H, Yoshino K, Yoshida K, Matsumura H, et al: **Large-scale gene disruption in Magnaporthe oryzae identifies MC69, a secreted protein required for infection by monocot and dicot fungal pathogens.** *PLoS Pathog* 2012, **8:**e1002711.

11. Zhong Z, Chen M, Lin L, Han Y, Bao J, Tang W, Lin L, Lin Y, Somai R, Lu L, et al: **Population genomic analysis of the rice blast fungus reveals specific events associated with expansion of three main clades.** *ISME J* 2018, **12:**1867-1878.

12. Chen S, Songkumarn P, Venu RC, Gowda M, Bellizzi M, Hu J, Liu W, Ebbole D, Meyers B, Mitchell T, Wang GL: **Identification and characterization of in planta-expressed secreted effector proteins from Magnaporthe oryzae that induce cell death in rice.** *Mol Plant Microbe Interact* 2013, **26:**191-202.

13. Guo X, Zhong D, Xie W, He Y, Zheng Y, Lin Y, Chen Z, Han Y, Tian D, Liu W, et al: **Functional Identification of Novel Cell Death-inducing Effector Proteins from Magnaporthe oryzae.** *Rice (N Y)* 2019, **12:**59.

14. Irieda H, Inoue Y, Mori M, Yamada K, Oshikawa Y, Saitoh H, Uemura A, Terauchi R, Kitakura S, Kosaka A, et al: **Conserved fungal effector suppresses PAMP-triggered immunity by targeting plant immune kinases.** *Proc Natl Acad Sci U S A* 2019, **116:**496-505.

15. Shimizu M, Nakano Y, Hirabuchi A, Yoshino K, Kobayashi M, Yamamoto K, Terauchi R, Saitoh H: **RNA-Seq of in planta-expressed Magnaporthe oryzae genes identifies MoSVP as a highly expressed gene required for pathogenicity at the initial stage of infection.** *Mol Plant Pathol* 2019, **20:**1682-1695.

16. Wang Y, Wu J, Kim SG, Tsuda K, Gupta R, Park SY, Kim ST, Kang KY: **Magnaporthe oryzae-Secreted Protein MSP1 Induces Cell Death and Elicits Defense Responses in Rice.** *Mol Plant Microbe Interact* 2016, **29:**299-312.

17. Kang S, Sweigard JA, Valent B: **The PWL host specificity gene family in the blast fungus Magnaporthe grisea.** *Mol Plant Microbe Interact* 1995, **8:**939-948.

18. Sweigard JA, Carroll AM, Kang S, Farrall L, Chumley FG, Valent B: **Identification, cloning, and characterization of PWL2, a gene for host species specificity in the rice blast fungus.** *Plant Cell* 1995, **7:**1221-1233.

19. Mentlak TA, Kombrink A, Shinya T, Ryder LS, Otomo I, Saitoh H, Terauchi R, Nishizawa Y, Shibuya N, Thomma BP, Talbot NJ: **Effector-mediated suppression of chitin-triggered immunity by magnaporthe oryzae is necessary for rice blast disease.** *Plant Cell* 2012, **24:**322-335.
