# Supplementary material for: Histone modification dynamics at H3K27 are associated with altered transcription of in planta induced genes in Magnaporthe oryzae
Source: PLoS Genet. 2021 Feb 3;17(2):e1009376. doi: 10.1371/journal.pgen.1009376 (PMC7886369; doi:10.1371/journal.pgen.1009376)
Supplement: Attachment — Submitted filename: PLOS_geneResponse_final.docx [file pgen.1009376.s029.docx]

Reviewer's Responses to Questions are shown in blue

**Comments to the Authors:
Please note here if the review is uploaded as an attachment.**

**Reviewer #1:** In this study, Zhang et al. describe genome wide histone modification dynamics (H3K27me3, H3K27ac and H3K36me3) under in vitro growth conditions in the plant pathogenic fungus Magnaporthe oryzae. The authors generate deletion mutants of several PRC2 components and the acetyltransferase Gcn5 to analyze genome wide H3K27 dynamics and their transcriptional implications in vitro and in planta. They find that transcriptional repression in vitro correlates to the presence of H3K27me3 and lack of H3K27ac. Loss of H3K27me3 in the kmt6 mutant results in de-repression and enrichment of H3K27ac in at least some of the regions. Among those de-repressed genes are known effectors and in planta induced genes. The manuscript has a clear structure and is well written.

Since in-planta ChIP was not performed, there is no proof of actual chromatin remodeling in planta but only a correlation between in vitro chromatin states and in planta transcription. This correlation has been shown before in other plant pathogens. Without additional ChIP-seq experiments in planta or at least comparable conditions, the findings in this manuscript do not provide significant advances over previous correlations of in planta induced genes and in vitro chromatin states.

Comments:

The PRC2 mutants show an altered phenotype upon growth on RPA and to a lesser extent on CM. What was the reason to choose CM as growth medium for ChIP and RNA-seq and not RPA? What stage in the life or infection cycle of Magnaporthe comes close to growth in CM?

Authors: Complete media (CM) was chosen as an *in vitro* growth condition that represents a different physiological state than *in planta* growth. We conducted our ChIP-seq and RNA-seq under these conditions to create a baseline condition on which to compare potential dynamics. Our RT-PCR and RNA-seq confirms this is accurate, as most genes important for infection are silent during CM culture. We have not conducted RNA-seq or ChIP-seq on Rice Polish Agar (RPA) as it is only made as a solid media. Fungal growth on solid media is currently not technically amenable to our RNA-seq and ChIP-seq protocols.

Since ChIP-seq in planta is challenging and to date there is no study that goes beyond correlation of ChIP-seq and RNA-seq data from different growth conditions, it would be great to show at least different in vitro conditions that result in chromatin and transcriptional changes. Is there any kind of growth media (minimal medium, containing rice leaves etc.) that alters the transcriptional program and would allow to detect chromatin changes? Although this would of course not be identical to a real plant infection, this could be an opportunity to detect local chromatin changes that reflect similar changes during infection.

Authors: From our experience and published literature, there is not a known growth media that mimics *in planta* conditions. To address the important question of *in planta* histone modification dynamics (i.e. ‘detect changing chromatin changes’), we performed *in planta* ChIP-qPCR for the gene body and promoter using four *M. oryzae* loci. These experiments are technically difficult and produced limited amounts of DNA, but our results show both H3K27me3 and H3K27ac changes *in planta*. The revised manuscript contains an updated results section including Figure 7, updated materials and methods, and an updated discussion.

Removal of all H3K27me3 like in the kmt6 mutant is an highly artificial chromatin state that likely has many effects on expression that are not comparable to local chromatin remodeling during plant infection.

Authors: We agree with the authors comment that genetic perturbation likely results in non-identical dynamics to those seen under natural conditions. However, the presented results test specific aspects of histone modification dynamics and transcriptional response, and the results support the hypothesis that this type of regulation contributes to expression of *in planta* genes. These results are further supported from the *in planta* ChIP-qPCR (Figure 7) that has been added to the revision.

Does deletion of kmt6 or gcn5 affect pathogenicity on the plant?

Authors: We conducted infection assays and have included the results in supplemental figure 14 in the revision. We found that both the *kmt6* and *gcn5* KOs are able to infect the plant, but they produce smaller lesions. The double mutant (*kmt6/gcn5*) has a severely affected growth and is not able to infect rice plants. The revised manuscript has an updated results, discussion, and materials and methods to reflect this data.

Are the differences in signal intensity of H3K27me3 on TEs correlated to copy number or mapping of reads to TEs?
Authors: The signal intensities come from the MACS2 peak calling algorithm, which normalizes the ‘signal’ intensities across the replicated ChIP libraries and also relative to the ‘input’ ChIP-seq control libraries. Each TE in the genome therefore gets a normalized intensity and should be the result of read mapping and not TE number. We can confirm this because the number of TEs for the different families shown in supplemental figure 2B are very different, yet the signal intensities are not significantly different. For example, there are 1170 Gypsy elements, 588 Tad1 elements, and 183 CRE elements with an H3K27me3 peak. All three families have a similar signal intensity despite having a wide range of copy number in the genome. We have updated supplemental figure 2B to show the number of elements in each family to make this point more clear.

In Fig 3B it appears that genes that are normally marked by H3K27me3 in WT, gain H3K27ac in the kmt6 mutant. Conversely, ~ 10 % of non-marked genes lose H3K27ac signal. What kind of genes lost H3K27ac in the kmt6 mutant and does this correlate to the expression profile?

Authors: We updated the results section and added supplemental figure 9 to detail the transcriptional impact of genes losing or gaining H3K27ac. We found that genes gaining H3K27ac had significantly higher transcription, while genes that lost it remained the same. We used GO and KEGG to identify that H3K27me3 marked genes are mainly involved in primary and secondary metabolism (supplemental table 4 and 5). We did not find any specific association (GO/KEGG) for genes that gained or lost acetylation in *ΔMokmt6* and have not added these results to the revision as they do not impact the findings.

How was the percentage of marked genes or TEs determined? Overlap or proximity of peaks and genes or promoters?
Authors: Genes and TEs were defined as marked by a histone modification if the ChIP peak, as determined from MAC2, overlapped >50% of the gene/TE. The boundaries for the genes and TEs were defined as the transcription start and stop site for genes and the boundaries for TEs. This information has been more explicitly referenced in the methods section of the revision.

Are specific functional gene categories enriched among H3K27me3 marked genes?

Authors: We ran Blast2GO, but there was not an enrichment for a GO term. This is likely because many genes are ‘unknown function’ and have no GO term associated. We also used KEGG and identified that many H3K27me3 marked genes are involved in primary and secondary metabolism. These results are added to the revision along with supplemental table S4 and supplemental table S5.

Is there a difference between Figure 3C and S7A?

Authors: Figure S7A is the same as Fig 3C, but was included in supplementary figure 7 for comparison. We have removed figure S7A in the revision to avoid confusion.

Since Gcn5 is not only responsible for acetylation of H3K27 but many other lysines on different histone tails and also interacts with RNA polymerase II, it is very difficult to determine if presence/absence of H3K27ac has any functional consequences. There may very well be a correlation to the transcriptional state of the locus but there is little evidence that H3K27ac alone has functional implications here.

Authors: We agree that the function of GCN5 is very broad. Our functional data regarding *gcn5* mutation could be caused by a loss of H3K27ac or alternatively because of other GCN5 effects (i.e. pleiotropy). Our results show a correlation between H3K27ac and transcription, but it does not prove H3K27ac is sufficient for transcriptional activation. We have updated the title of our manuscript and our discussion section in the revision to more clearly acknowledge this.

To better understand dynamics of Gcn5 and H3K27me3, a single deletion mutant of Gcn5 and ChIP-seq profiles of H3K27me3 would be a relevant addition.
Authors: As the reviewer commented, GCN5 has many functions. We specifically conducted research using the *gcn5/kmt6* double mutant to assess the hypothesis that loss of H3K27me3 allowed gain of H3K27ac and results in increased expression. We believe that single *gcn5* KO will not produce interpretable data regarding our hypotheses. The data are likely to show that many genes are differential expressed and have altered ChIP-seq profiles, but given the pleiotropic effects, interpretation is difficult. As such, we decline to spend the time and money to conduct these experiments as they will not contribute to the results of our research.

Are the 27 (presumed) effectors all known or predicted effectors in Magnaporthe? If there are more predicted effector, how many are marked by H3K27me3?

Authors: We chose the list of 27 effectors based on each one having published functional characterization and being present in our study strain, Guy11. That is, these 27 genes represent high-confidence effectors, well studied *in planta* induced genes or known avirulence genes (Supplemental Table S9). In the revised manuscript, we added results for presumed effectors identified using the EffectorP software (https://github.com/JanaSperschneider/EffectorP-2.0). The candidate effectors from this software, including those we could corroborate with empirical evidence, were significantly enriched for H3K27me3 (added supplemental Table S7 and S8). The results section and materials and methods have been updated in the revision.

Figure 6B: Since there is no in planta ChIP-seq data available, ChIP qPCR of candidate genes *in planta* would give an idea about changing chromatin states here.

Authors: We conducted ChIP-qPCR from replicated infected plants. These are difficult experiments and do not provide much material to conduct experiments, never the less, we have succeeded in generating high-quality data. We identified that H3K27me3 was significantly reduced at the promoter of one of the tested effectors, but H3K27me3 levels were generally stable across the tested gene body locations. Details for these experiments have been added to the results, materials and methods and discussion section, as well as the addition of figure 7.

Line 284: I don’t think it is correct to compare activation rate in a very small selected subset of genes to the genome-wide number of upregulated genes in the kmt6 mutant.

Authors: The analysis in question is a chi-square test to determine if there is an association between two variables, namely being a high-confidence effector and being up-regulated in Δ*Mokmt6*. While the number of genes in the high-confidence effector list is low, it does not result in any violations of the assumptions for a chi-square test. That is, all of the cells of the 2x2 contingency table have values > 5. We have updated the revision by adding supplemental tables 7, 8, 10 and 11, which lists the 2x2 contingency tables for the various chi-square tests run in this section of the results allowing the reader to clearly see what was tested.

**Reviewer #2**: In this work, Zhang and colleagues describe histone modification dynamics at H3K27 site regulate gene transcriptional landscape. In particular, those in planta induced genes are subjected to H3K27me3/H3K27ac regulation in Magnaporthe oryzae. The authors showed that H3K27me3 provides significant local transcriptional repression and contributes to normal in vitro growth by deleting and complementing the H3K27me3 methyltransferase complex PRC2 core components. Moreover, H3K27ac was redistributed to previously occupied H3K27me3 sites, was required for transcriptional activation of roughly a third of differentially expressed genes. The authors also observed that many in planta induced genes were marked by H3K27me3, and detailed how altered H3K27 modification influences transcription of in planta induced genes. The data is carefully analyzed and presented, and I am fine with their statement of the major conclusion. Overall, this is an interesting paper that merit the publication. However, it seems to me that the story still has holes and I hope they could address these issues.

Major concerns:
(1) Regarding that M. oryzae is an important rice pathogen and gene expression pattern of in planta induced genes are impaired in mutants. The paper didn’t show infection results, what about the pathogenicity of different M. oryzae mutants that are used in this research?

Authors: As responded to reviewer #1, we conducted infection experiments with the mutant lines. We find that both single mutants are able to infect, but produced smaller lesions. The double mutant has a significantly reduced growth and morphology and is not able to infect rice. These results have been added along with supplemental figure 13 and 14.

(2) The authors found histone modifications at H3K27 contribute to regulation of genes important during host infection. For example, many effector genes were up-regulated in H3K27me3 deficient mutants. What about the H3K27me3 and H3K27ac level of these induced genes during infection in WT and mutants? These important data are missing in the current manuscript. Without these data, it is hard to set up a link between gene expression and the modification changes. I suggest authors to show a few cases by adding a few experimental data.

Authors: As noted by the reviewers, there are no genome-wide *in planta* ChIP experiments published due to technical limitations. We did conduct ChIP-qPCR (Figure 7) from infected rice leaf sheaths and were able to generate very high-quality data (see response above). These results have been added to the revision in several places.

other concerns:
(1) The authors observed that H3K27ac was negatively correlated with H3K27me3(r=-0.61, p < 2.2e-16) and H3K36me3 was positively correlated with H3K27me3(r= 0.61, p < 2.2e-16). H3K36me is well known as a activate mark, is H3K36me3 a repressive mark in M. oryzae? What about the expression change of genes that lost H3K36me3 in Mokmt6 mutant?

Authors: We have added supplemental figures 9, 10 and 11 to further detail the impact altered H3K27ac and H3K36me3 levels have on gene transcription. Our results do support that H3K36me3 is associated with repressed transcription, but likely only when it is associated with regions marked by H3K27me3. This is consistent with recent reports in *Fusarium fujikuroi* (*Janevska, 2018, genetics*) and *Neurospora crassa* (*Bicocca, 2018, elife*), where the authors suggest that H3K36me3 has different functions across the genome. We have updated our manuscript’s results and discussion to integrate these findings.

(2) Do some genomic regions gain H3K27ac but lost H3K36me3 at the same time? What about these genes expression change compare to genes gained H3K27ac or lost H3K36me3 only?

Authors: We identified the genes that either gain H3K27ac, lose H3K36me3, or both and added these results in supplemental figure 11. The results show expression changes in *ΔMokmt6* which are consistent with our analysis, where these genes on average have increased expression when they gained H3K27ac or lost H3K36me3.

(3) GCN5 can acetylate H3K9ac, H3K14ac, H3K18ac, H3K23ac, H3K27ac and so on, the authors found many H3K27me3 marked genes activated by H3K27ac, how to exclude crosstalk of H3K27me3 and other histone acetylation?

Author: We cannot exclude crosstalk between H3K27me3 and acetylation of other histone modifications. Nevertheless, the results presented in our manuscript offer a detailed and comprehensive analysis regarding dynamics at H3K27 and transcriptional regulation. We have updated our discussion section to more clearly state the limitations of our work and highlight alternative explanations (i.e. hypotheses).

(4) In fig6, the authors analyzed 27 effector gene that are presented in M. oryzae strain Guy11. What about other secreted proteins? I assumed that M. oryzae should have many effector genes.

Authors: Indeed, *M. oryzae* has many putative effectors. We have updated our results to include analysis of putative effectors identified with effectorP. We originally included only the 27 effectors/avirulence genes that have experimental evidence (Supplemental Table S9), as we think this is the highest confidence set of genes. We similarly highlighted the set of effectorP candidates that actually show differential expression during in planta growth. We again see that these genes are statistically more highly associated with H3K27me3 than would be expected by chance. These results have been added to the revised manuscript.

(5) Many in planta induced genes were up-regulated in ΔMokmt6, what about the expression change of these genes in ΔMokmt6 during infection?

Authors: We have conducted infection experiments as detailed in other responses, and added the results to the revision. The *ΔMokmt6* line has an altered infection profile compared to WT (i.e. less virulent). Due to this, and the fact that ~10% of the genome is DE in Δ*Mokmt6* , we believe it is difficult to make precise conclusions from this infection data because of the pleiotropic effects of *ΔMokmt6*. Our analysis motivation was in response to the specific hypothesis that H3K27 modification dynamics plays a role in regulating *in planta* expressed genes. We do not claim that this is the only function for H3K27me3/ac. As such, we have not conducted expression analysis of the mutant infecting a plant because it will not be possible to interpret the data.

**Reviewer #3**: Understanding how genes from pathogenic and endophytic fungi are specifically upregulated/derepressed in planta remains an important question to be answered. But what is clear from work published to date is the important role specific histone marks play in controlling chromatin structure and gene expression in planta. An important limitation to addressing this question is the technical difficulty in studying histone modification dynamics at a global scale during plant infection as noted by the authors in the Discussion of this manuscript. Working within these constraints the authors report on some important new insights into the role of chromatin structure changes in the plant pathogen Magnaporthe oryzae by specifically focusing on the dynamics of histone H3K27 modification. While much of the work examines changes that occur ex planta, which uncovers good information about the repressive state of plant induced genes, the authors are still able to provide some new insights into what might be happening in the plant by aligning global changes in histone dynamics in axenic culture with in planta transcriptomics using wild type and mutants defective in H3K27 methylation or acetylation. The work is of a high technical standard and a very important contribution to the growing literature in this field.

ChIP analysis of H3K27me3 and H3K27ac signals across the genome (Fig 1) shows that these two marks have distinct distribution profiles with a predominance of the former in regions of the genome enriched with TE of the Class I type. The H3K27ac were almost exclusively associated with genes actively transcribed in axenic culture. These experiments provide important genome scale insights into the distribution of these two histone marks and highlight the importance of H3K27me3 as a repressive mark.

Authors: Thank you for your positive comments and we agree this work will add to the body of literature on this topic.

The authors then go onto show by deletion analysis that three components of the PRC2 complex are required for H3K27 trimethylation with a statistically significant effect on vegetative growth (Fig 2).

Using the kmt6 deletion mutant they go on to show that loss of H3K27me3 results in a specific redistribution of H3K27ac marks (Fig 3). However, as highlighted in the Discussion the mechanism underlying this redistribution is not clear. Interestingly, they found that H3K36 methylation marks correlate with H3K27me3 marks, and these are lost in the kmt6 mutant, providing good evidence for cross talk between these two methylation modifications. The text in this section (around line 159) could be clearer by specifically mentioning whether they are looking at changes in marks in WT or mutant.

Authors: We have updated the text in the revision for this section of the results to clarify between the wild type and mutant with regards to changing histone modification levels.

The subsequent cluster analysis used to examine more deeply the chromatin dynamics provides further support for reciprocity between H3K27me3 and H3K27ac and cross talk with H3K36me3. However, the difference in colour coding between the upper and lower panels of Fig 3C does make it difficult for the reader to interpret this Figure – there is either an error here or I have not understood the difference?

Authors: We realize that our coloring for the words ‘cluster 1 and cluster 2’ and the line color for the top panel of figure 3C was confusing as it was a similar color to the heatmap. We modified these colors to improve the interpretation.

RNAseq is then used to examine transcriptome differences between WT and the kmt6 mutant and between WT and the kmt6/gcn5 double mutant. The high percentage of the upregulated genes (87.9%) in the mutant that have H3K27me3 marks provides strong evidence that the Kmt6 H3K27 methylase is required for the H3K27 methylation observed and gene repression. However, the evidence that acetylation is required for expression at these gene loci was not so strong given only a third of the K27me3 marked genes that were upregulated in the kmt6 mutant were downregulated in the kmt6/gcn5 double mutant. I think it is important for the culture phenotype of the double mutant to be reported as I suspect the double mutant will impact on growth and development.

Authors: We have updated the results section to include analysis of the double kmt6/gcn5 mutant. Indeed, it has a reduced growth rate, altered spore morphology and is no longer pathogenic to rice. These results were added to the revision and can be seen in supplemental figure 13 and 14.

Also, a little more information on the specificity of the gcn5 gene product is needed. I note in the discussion it is described as a general acetyl transferase but in the results section it is said to be the major histone acetyl transferase for H3K27 acetylation.

Authors: We have updated the results and discussion to clarify this point. GCN5 is the enzyme responsible for H3K27ac, but this is not the only lysine it is able to acetylate.

The integration of the RNAseq data with ChIP-seq data provides further support for the connection between the H3K27 modification status and gene expression – very nice work.

Authors: Thank you for your comment.

The final two sections of the Results attempt to bridge the gap between what is observed in axenic culture and what might be happening during plant infection. Having identified the H3K27 marked genes in the earlier experiments the authors next examine how the expression of these genes changes in planta. Of the ~1000 genes differentially expressed in planta compared to axenic culture 44% of these fall within the H3K27 marked genes further highlighting the importance of this mark in controlling gene expression between the two physiological conditions. Two thirds of these H3K27 marked genes were up-regulated in the kmt6 mutant. Taken together this section highlights the importance of this mark for repression of this subset of genes in culture and the need for removal of this mark for high level of expression in planta. In presenting this work I would discourage the authors from talking about genes being up-regulated in planta when the comparison is with expression in culture which is a totally different and discontinuous physiological condition. I think it is acceptable to talk about up-regulation when comparing gene expression in culture for mutant and WT.

Authors: We have modified the text in the results section to clarify that *in vitro* and *in planta* growth cause very different physiological responses, and refer to differences in transcriptional levels.

Finally, the authors hone in on the genes encoding known and putative effectors in M. oryzae. I was surprised that there are just 27 known or presumed effectors present in M. oryzae?

Authors: We have modified the text to clarify that these are not the only presumed/candidate effectors, but these 27 are the ones for which there are published experimental results (Supplemental Table S9). We felt this was a much higher confidence list than one from a prediction software, but we have performed candidate effector identification with effectorP. The results on the broader possible candidate effector list have been included in the results.

This seems a very small number compared to the numbers identified in other fungal plant pathogens. While this analysis does highlight the potential importance of H3K27 modifications in regulating the chromatin state and expression of some effector genes in planta the regulation of others is clearly more complex. This is perhaps not surprising given this group of genes will be required at different stages of infection during colonization of the host. I thought the hierarchical clustering approach used for this analysis was good. Although the results are complex they do highlight the important role H3K27methylation plays in controlling expression of plant-induced genes. One important limitation of comparing gene expression in culture with that in planta and drawing conclusions about the chromatin state that should be highlighted is the growth conditions used ex planta are invariably unlike what will be found in planta. This is an important physiological limitation than many papers fail to mention.

Authors: We have updated the discussion section to explicitly acknowledge that our data supports the proposed hypothesis, and acknowledge the limitation of extrapolating our findings from axenic growth to *in planta* conditions. We believe the results add significant information to the study of fungal gene regulation as acknowledged by the reviewers.

In summary, this is a comprehensive piece of work carried out to a high technical standard that provides important new insights into the role of histone modification dynamics in controlling gene expression in the plant pathogen M. oryzae. As discussed above there are some major technical challenges in examining these dynamics in planta but despite this limitation this work increases our understanding of the importance of the H3K27 modifications in controlling gene expression.
